# Pupillary response reflects attentional modulation to sound after emotional arousal

**DOI:** 10.1038/s41598-021-96643-7

**Published:** 2021-08-26

**Authors:** Satoshi Nakakoga, Kengo Shimizu, Junya Muramatsu, Takashi Kitagawa, Shigeki Nakauchi, Tetsuto Minami

**Affiliations:** 1 Department of Computer Science and Engineering, Toyohashi University of Technology, 1-1, Hibarigaoka Tempaku, Toyohashi, Aichi 441-8580 Japan; 2 System & Electronics Engineering Dept. II, TOYOTA Central R&D Labs., Inc., 41-1, Yokomichi, Nagakute, Aichi 480-1192 Japan; 3 R&D and Engineering Management Div., TOYOTA MOTOR CORPORATION, 1, Toyota-cho, Toyota, Aichi 471-8502 Japan; 4 Electronics-Inspired Interdisciplinary Research Institute, Toyohashi University of Technology, 1-1, Hibarigaoka Tempaku, Toyohashi, Aichi 441-8580 Japan

**Keywords:** Psychophysics, Neurophysiology

## Abstract

There have been various studies on the effects of emotional visual processing on subsequent non-emotional auditory stimuli. A previous study with EEG has shown that responses to deviant sounds presented after presenting negative pictures collected more attentional resources than those for neutral pictures. To investigate such a compelling between emotional and cognitive processing, this study aimed to examined pupillary responses to an auditory stimulus after a positive, negative, or neutral emotional state was elicited by an emotional image. An emotional image was followed by a beep sound that was either repetitive or unexpected, and the pupillary dilation was measured. As a result, we found that the early component of the pupillary response to the beep sound was larger for negative and positive emotional states than the neutral emotional state, whereas the late component was larger for the positive emotional state than the negative and neutral emotional states. In addition, the peak latency of the pupillary response was earlier for negative than neutral or positive images. Further, to compensate for the disadvantage of low-temporal resolution of the pupillary data, the pupillary responses were deconvoluted and used in the analysis. The deconvolution analysis of pupillary responses confirmed that the responses to beep sound were more likely to be modulated by the emotional state rather than being influenced by the short presentation interval between the images and sounds. These findings suggested that pupil size index modulations in the compelling situation between emotional and cognitive processing.

## Introduction

Most often, an emotional state modulates the processing of subsequent stimulus inputs to the senses. For example, various studies have shown that emotional visual stimuli modulate subsequent processing of non-emotional auditory stimuli^1^. It is known that the blink magnitude in response to unexpected sounds after the presentation of negative pictures was enhanced; whereas, that after the presentation of positive pictures dampened^2^. A study on electroencephalogram event-related potentials (ERPs) has also shown that the response to acoustic startle probes (P3 component) is modulated by emotionally arousing pictures in the foreground^3^. In terms of human evolution, these phenomena result from adaptation to the transfer of enhanced attention between modalities in a dangerous environment.

Some studies using two types of probe sounds (standard tones, which are presented frequently or as part of a repetitive background, and oddball tones, which are presented infrequently and thus are considered deviant) have shown that the response to sound deviance is modulated after emotional picture presentation^4–6^. In these studies, the auditory stimuli are task-irrelevant. In contrast, some of the subsequent auditory stimuli are task-relevant. For example, Tartar et al. investigated the ERPs associated with two types of beeps (standard and oddball tones) that were presented after viewing negative or neutral images^7^. The results in N1 and N2, which reflect early auditory processing, showed that the amplitude of the beep presented after negative visual stimuli were enhanced regardless of the frequency of the beeps. As for the LPP (late processing negativity), which reflects late auditory processing, there was no difference between the negative and neutral pictures presented before the standard beep. However, in the oddball tone condition, the LPP was amplified by negative pictures than neutral pictures. These results were interpreted as increased perceptual resources for all sensory modalities when emotion was induced by the pictures^8^. In addition, the oddball tones shared the increased attentional resources activated by the negative images, and late processing negativities were enhanced by automatic attention^9^. However, this previous study focused only on the negative state, and the response to subsequent tone stimuli of different presentation frequencies in the positive state has not yet been clarified.

In this study, we used pupillary response as a physiological index that reflects emotional and attentional states to subsequent tone stimuli. Pupillary responses are less expensive to measure and have recently received more attention. The Pupillary Dilation Response (PDR) is controlled by the level of activity of the locus coeruleus (LC)^10^. Therefore, the pupillary response has been considered indirect evidence of mental activity such as arousal; Bradley et al. found that the pupillary response to images from the International Affective Picture Set (IAPS)^11^ was associated with the arousal level of the emotional stimuli, suggesting that the pupil is mediated by sympathetic nervous system activation^12^. Many studies investigating the association between emotionally arousing stimuli, such as images and sounds, and pupillary responses have reported that participants show more pupillary dilation to high arousal stimulus^13–18^. In addition, the pupil diameter, as well as the EEG P3 component, reflects the change in task engagement predicted by the Adaptive Gain theory of LC-NE function during the oddball task^19^.

This study investigated the response to tone stimuli with different presentation frequencies (standard and oddball) after the presentation of emotionally arousing pictures using pupillometry. If the affective stimulus modulates the attentional state to the subsequently presented stimulus, then the pupil response to the tone stimulus, which is unrelated to the affective stimulus, would be affected by the arousal and valence of the prior affective stimulus. As the index of pupillary activity, we used the early and late components as well as the peak latency of the PDR after tone presentation. We hypothesized that negative states would elicit greater PDRs to oddball sounds than neutral states. In addition, we investigated the responses to tone stimuli in three emotional states: positive, negative, and neutral. Many studies suggest that the pupillary response to emotional stimuli reflect the arousal level regardless of the emotional valence^13,20^. However, it is not clear whether the pupil response to subsequent stimuli after emotional stimuli is related to its arousal or valence^6,21–23^. We hypothesized that the negativity bias would lead to an earlier latency of PDRs for beep sounds in the negative state than positive and neutral states. As the interval between the presentation of the emotional picture and beep sound was short (600 ms), we separated the responses to the two stimuli using high-resolution deconvolution analysis by Wierda et al.^24^.

## Results

Thirty-two students (23 males; mean age = 21.3 years; range = 20–24 years; SD = 1.14 years) participated in this experiment. In this study, we investigated the pupillary response to tone after the presentation of the emotional picture. Participants were presented with an emotional picture (positive, negative, or neutral) followed by two types of tone (standard or oddball). The participants responded to the combination of pictures and tones, and their pupillary responses were measured as a physiological indicator.

### Behavioral response

**Figure 1:**
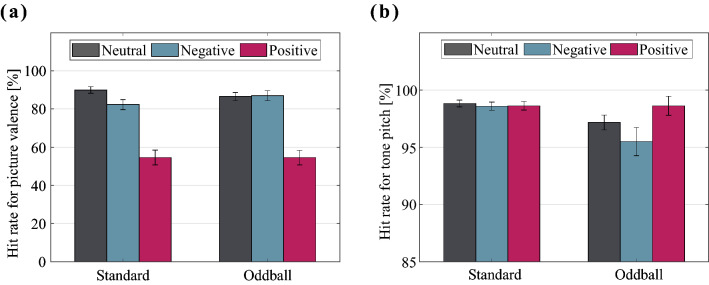
Behavioral results. (**a**) The hit rates for the picture valence. (**b**) The hit rates for the tone pitch. The error bars indicate the standard error of the mean.

The behavioral data are presented in Fig. 1. Figure 1a shows the hit rates for the picture valence) for each sound frequency and emotion condition. Behavioral analysis of the hit rates revealed a main effect of picture category ($F\left(1.83, 53.05\right)=49.919, \, p<0.001, \, {\eta }^{2}=0.633$), but no main effect of tone type ($F\left(1, 29\right)=0.041, \, p=0.840, \, {\eta }^{2}=0.001$). In addition, the picture category $\times$ tone type interaction was significant ($F\left(1.77, 51.31\right)=3.938, \, p=0.030, \, {\eta }^{2}=0.120$). Post hoc analyses revealed that the hit rates were significantly higher for a standard tone than an oddball tone when it followed a neutral picture ($F\left(1, 29\right)=4.679, \, p<0.039, \, {\eta }^{2}=0.139$), and a negative picture ($F\left(1, 29\right)=6.639, \, p=0.015, \, {\eta }^{2}=0.186$). Additionally, there was a main effect of picture category for standard tones ($F\left(1.72, 49.89\right)=46.838, \, p<0.001, \, {\eta }^{2}=0.618$) and a main effect of picture category for oddball tones ($F\left(1.92, 55.8\right)=41.037, \, p<0.001, \, {\eta }^{2}=0.586$). Multiple comparisons for the picture category with a standard tone showed that the hit rates for positive images were lower compared to those for neutral and negative images $p<0.001$), and the hit rates for neutral images were higher than those for negative images for standard tone trials $p=0.036$). Multiple comparisons for the picture category with an oddball tone showed that the hit rates to positive images were lower compared to those for neutral and negative images (all $p<0.001$). Figure 1b shows the hit rates for the tone pitch for each sound frequency and emotional condition. The analysis of the hit rates revealed a main effect of tone type ($F\left(1, 29\right)=7.966, \, p=0.009, \, {\eta }^{2}=0.216$), which indicated that the hit rates for a standard tone were significantly higher than those for an oddball tone. However, there was no main effect of picture category ($F\left(1.88, 54.6\right)=1.299, \, p=0.280, \, {\eta }^{2}=0.043).$ There was no significant interaction ($F\left(1.86, 54.05\right)=0.855, \, p=0.424, \, {\eta }^{2}=0.029$).

### Pupillary response

**Figure 2:**
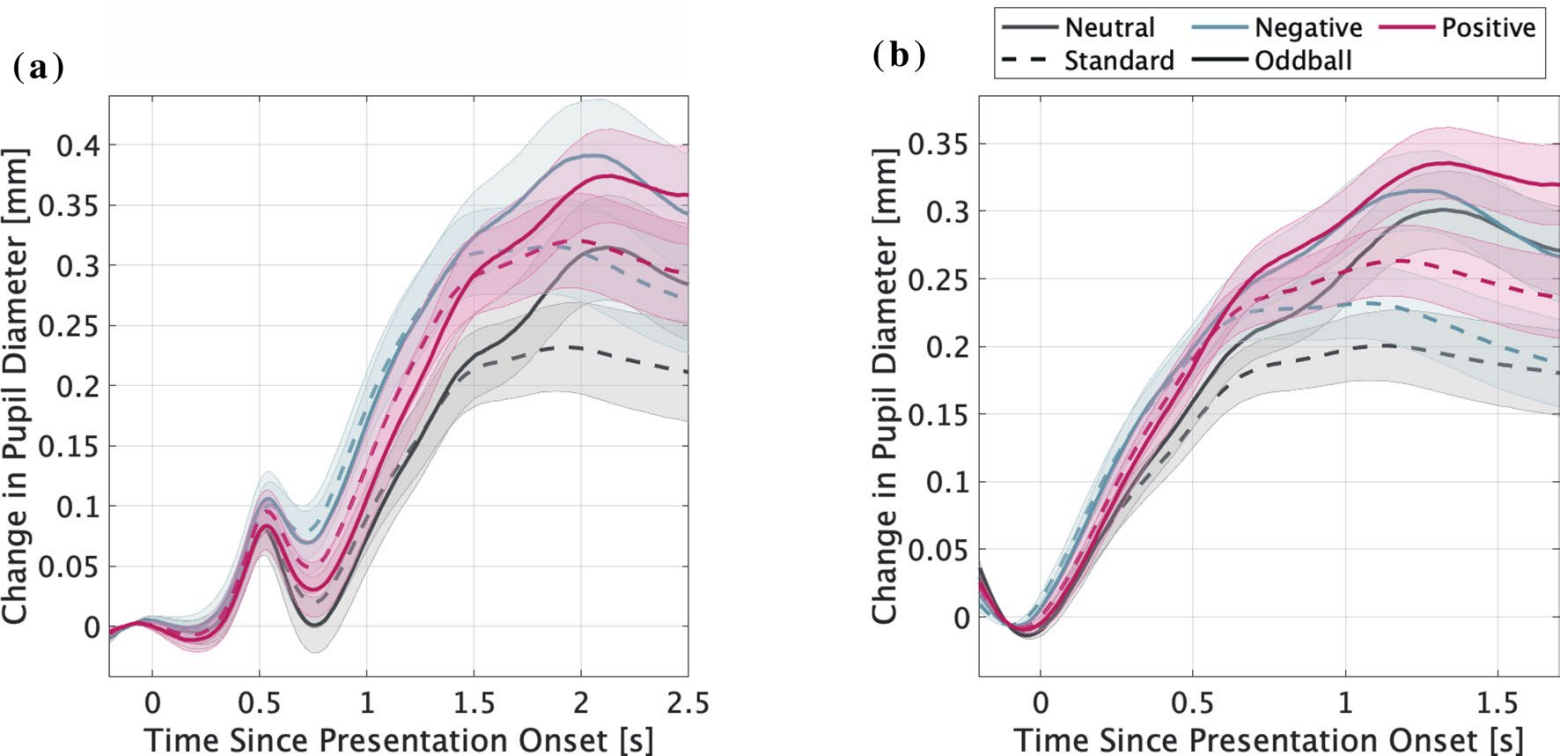
The grand-averaged time course of pupillary responses to the beep sounds (standard and oddball) in each picture category. (**a**) Pupillary responses using picture presentation onset as a baseline. (**b**) Pupillary responses using beep sound presentation onset as a baseline. The horizontal axis indicates the time (s), while the vertical axis indicates the grand-averaged change in pupil dilation from baseline (− 200 ms to 0 ms). Shaded areas represent the standard error of the mean.

**Figure 3:**
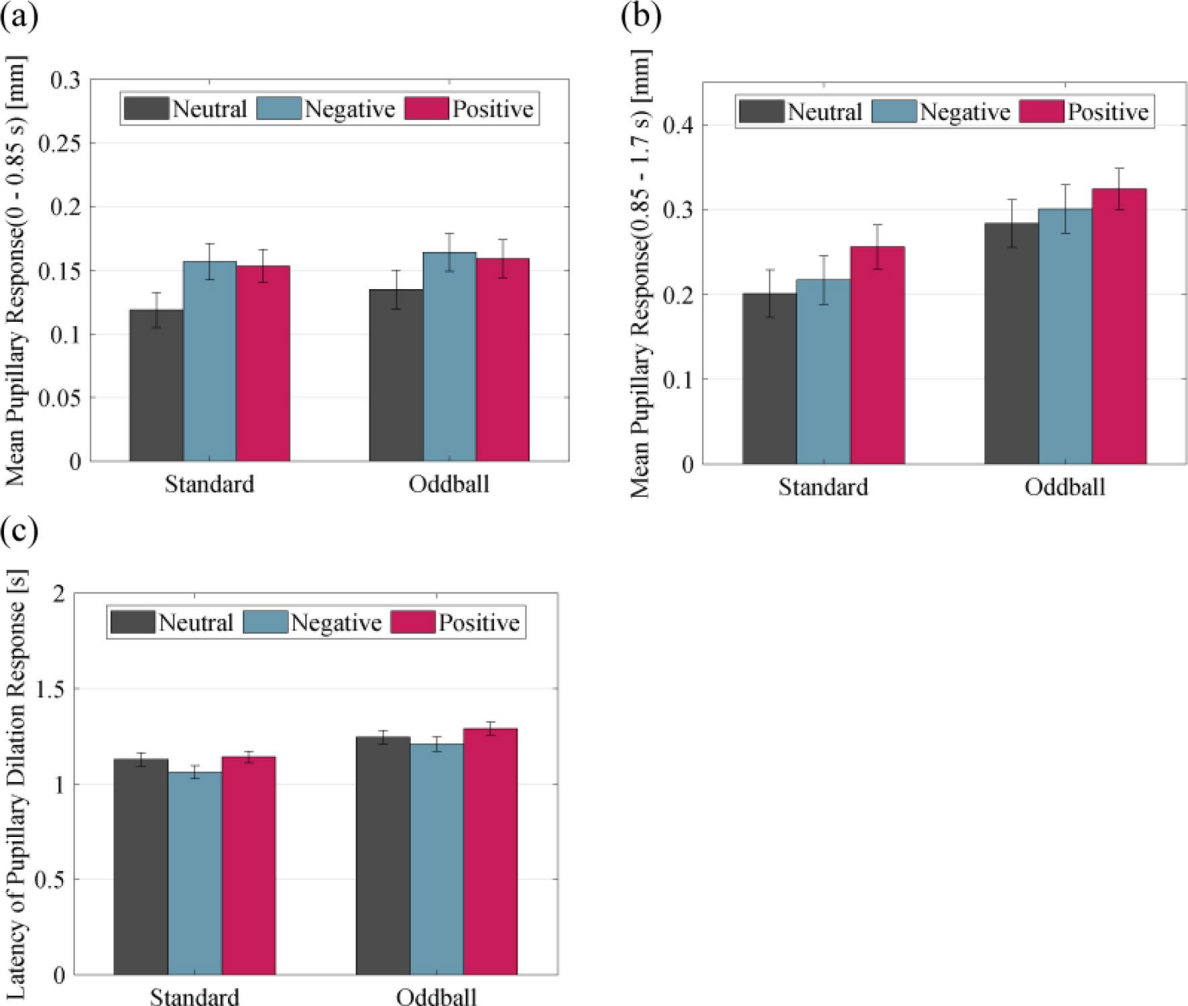
The pupillary dilation response (PDR) components. (**a**) The early component (mean PDR between 0 and 0.85 s). (**b**) The late component (mean PDR between 0.85 and 1.7 s). (**c**) The PDR latency between tone onset and peak PDR amplitude. The error bars indicate the standard error of the mean.

Figure 2 presents the grand-averaged pupil responses from the onset of picture presentation (Fig. 2a) and beep sound presentation (Fig. 2b), separated by picture category and tone type. The early component, late component, and PDR latency are shown in Fig. 3. The analysis of the early component revealed main effects of picture category ($F\left(1.82, 52.81\right)=14.670, \, p<0.001, \, {\eta }^{2}=0.336$). Multiple comparisons for picture category showed that the early component for negative and positive picture trials was larger compared to that for neutral picture trials (all $p<0.001$). In addition, there were significant main effects of tone type ($F\left(1, 29\right)=6.456, \, p=0.017, \, {\eta }^{2}=0.182$), which indicated the late component for standard tones was larger than that for oddball tones. There was no significant interaction ($F\left(1.84, 53.45\right)=0.766, \, p=0.460, \, {\eta }^{2}=0.026$).

The analysis of the late component revealed main effects for the picture category ($F\left(1.7 , 49.43\right)=15.826, \, p<0.001, \, {\eta }^{2}=0.353$). Multiple comparisons for picture category showed that the late component was larger for positive pictures compared to that for neutral and negative pictures (all $p<0.001$). In addition, there were significant main effects of tone type ($F\left(1, 29\right)=57.630, \, p<0.001, \, {\eta }^{2}=0.665$), which indicated that the late component for oddball tones was larger than that for standard tones. There was no significant interaction ($F\left(1.69, 48.87\right)=0.510, \, p=0.573, \, {\eta }^{2}=0.017$).

The analysis of the PDR latency revealed main effects of the picture category ($F\left(1.59 , 46.01\right)=11.851, \, p<0.001, \, {\eta }^{2}=0.290$). Multiple comparisons for the picture category showed that the PDR latency for negative pictures was shorter compared to that for neutral ($p<0.05$) and positive ($p<0.001$) pictures. In addition, there were significant main effects for the tone type ($F\left(1, 29\right)=55.236, \, p<0.001, \, {\eta }^{2}=0.656$), which indicated that the PDR latency was shorter for standard tones than oddball tones. There was no significant interaction ($F\left(1.86, 54.08\right)=0.729, \, p=0.478, \, {\eta }^{2}=0.025$).

**Figure 4:**
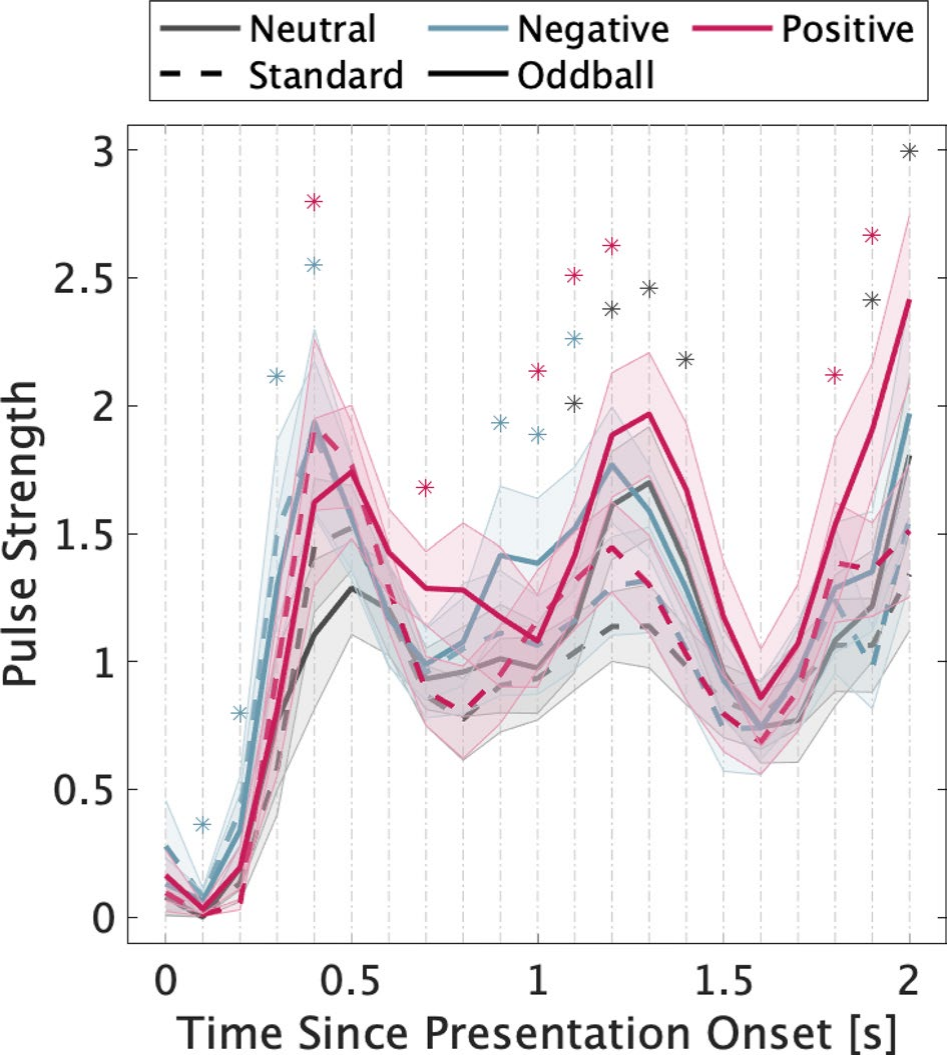
Deconvolved pulses for pupillary responses from the presentation onset of the picture. Shaded areas represent the standard error of the mean. Significant differences (α = 0.05) in the strength of the pulses are denoted by the asterisks. Red and blue asterisks indicate significant differences in the positive vs. neutral pictures and negative vs. neutral pictures comparisons, respectively (averaged within tone condition). Black asterisks indicate significant differences in the comparison of standard vs. oddball tones (averaged within picture category condition).

Figure 4 shows the deconvolved pulses from the pupil dilation. Table 1 indicates the results of t-tests for standard vs. oddball tones and emotional (positive or negative) vs. neutral pictures for each pulse. In the comparison between standard and oddball tones, the pulse strength of standard tones was higher than that of oddball tones at 1100–1400 ms and 1900–2000 ms after image presentation, respectively. In the comparison between neutral and negative images, the pulse strength of negative images was higher than that of neutral images at 100–400 ms and 900–1100 ms, respectively. In the comparison between neutral and positive images, the pulse strength of positive images was higher than that of neutral images at 400 ms, 700 ms, 1000–1200 ms, and 1800 ms–1900 ms.

**Table 1:** Results of t-test in time course data of the pulse strength.

Time (s)	Tone	Picture category	
	standard vs. oddball	neutral vs. negative	Neutral vs. positive
	p-value	p-value	p-value
0	0.309	0.097	0.109
0.1	0.951	0.015*	0.070
0.2	0.309	0.015*	0.406
0.3	0.599	0.011*	0.204
0.4	0.138	0.001*	0.003*
0.5	0.413	0.328	0.062
0.6	0.608	0.869	0.107
0.7	0.078	0.346	0.018*
0.8	0.052	0.152	0.242
0.9	0.112	0.030*	0.417
1.0	0.321	0.046*	0.044*
1.1	0.038*	0.033*	0.044*
1.2	0.005*	0.117	0.008*
1.3	0.002*	0.764	0.060
1.4	0.003*	0.883	0.216
1.5	0.059	0.492	0.420
1.6	0.683	0.802	0.956
1.7	0.833	0.345	0.212
1.8	0.647	0.218	0.033*
1.9	0.006*	0.897	0.016*
2.0	0.019*	0.534	0.083

0 s indicates the onset of picture presentation, and 0.8 s indicates the onset of tone presentation, respectively. Significant difference (α = 0.05) in the strength of the pulses are denoted by the asterisks.

## Discussion

We examined the pupillary responses to auditory stimuli in an oddball paradigm, with the auditory probe presented after images that arouse positive, negative, or neutral emotions to investigate the response to sound probe stimuli of different presentation frequencies during the emotional state. We found that the early component of the pupillary response was modulated by emotional arousal, while the peak latency of PDRs was shorter in the negative state than in the other emotional states. In addition, the results of the deconvolution analysis of pupillary responses indicated that the responses to beep sound were likely to be modulated by the emotional state rather than being influenced by the short presentation interval between images and sounds. Importantly, the frequency of sound stimulus presentation did not affect the modulation of pupillary response for each emotional state.

For the early component of the pupillary response, a main effect for the picture category was observed, and the pupillary responses to positive and negative images were larger than the responses to neutral images. This result is consistent with several previous studies, which reported that pupillary responses are associated with arousal regardless of valence because pupil dilation reflects arousal associated with increased sympathetic activity^12,13^. For the late component, a main effect for the picture category was also observed. In this case, the response to positive images was larger than the response to neutral or negative images. Taylor suggests an asymmetry in the negative state's emotional valence, positing that people in a negative emotional state make more effort to return to normal than those in a positive state^25^. In the present study, the late component of the PDR may have been smaller following negative images than positive images due to a decrease in the negative state arousal level as a result of the attempt to return to the normal state. In the early and late components of the pupillary response, a main effect was seen for the tone type, such that the pupil sizes were larger for deviant than for standard tones. Several studies measuring pupillary responses in the oddball stimuli have found greater PDRs about 1 s after deviant stimulus presentation than those in the standard stimuli^26–28^. This is consistent with the presence of PDRs due to deviant stimuli in the early and late components of the pupillary response in the present study. For the PDR latency, a main effect for the picture category was observed, and the peak latency of the response to negative images occurred earlier than that to neutral and positive images. Studies that have examined ERP in response to unrelated tonal stimuli presented during a state of emotional arousal have shown that the specific response to a tone in the negative state is found in the mismatch negativity (MMN)^6,23^ and P300a^21,22^ ERP components. Such effects are known as “negativity bias” and serve as priming for increasing attentional resources for the next stimulus^7^. Huang et al. examined the time course of negativity bias during processing by comparing multiple ERP components from early to late in each emotional state^29^. They reported that the negativity bias was expressed in both early and late processing stages, suggesting that the neural basis of both automated and controlled processes was involved in negativity bias. In this study, the finding that the peak latency of PDRs was earlier only during the negative state might reflect the aspect of automatic processing for the negativity bias, in that it increased with attentional resources to subsequent tone stimuli. However, a previous study showed that the later ERP's negativity bias was caused by the utilization of a specific category of less evocative pleasant stimuli^30^. Further research that considers emotional content will be needed to confirm that our results are related to negativity bias.

Further, we attempted to separate the pupillary responses to the two stimuli by deconvolution. The interval between image and sound presentation was short. The results showed a significant difference between the sound frequency conditions (Standard vs. Oddball) at 1.1–1.4 s and 1.9–2.0 s after image presentation. This difference is consistent with the results of the late component of the pupillary response to sound in Fig. 2b. In addition, the pupillary response to the deviant sound was also observed in the deconvolution pulse during image onset^24^. Considering this delay, significant differences between the image category conditions occurred about 0.9 s after the image presentation (0.1 s after the sound presentation), suggesting that these differences reflect pupillary responses to sound stimuli modulated by emotional states rather than responses to a previously presented image.

The present results provide evidence that the PDR reflects an oddball response. However, there was no interaction between the affective and sound presentation frequency conditions, and the frequency of presentation of the probe stimulus did not affect the modulation of pupillary response for each emotional state. Tartar et al. have compared ERPs for tones (standard vs. deviant) after presenting emotional pictures^7^. They reported that when the early components (N1 and N2) of the onset of the tone presentation were compared across emotional conditions, those for the negative were greater than those for neutral pictures in both standard and oddball tones. However, in the subsequent late processing negativity (LPP), which reflected late auditory processing, the amplitude was modulated by the emotional state only in oddball tones. If the pupillary response is reflected in the early automatic processing of attentional resources for auditory stimuli as described above, the reason for non-significant interaction between sound frequency and picture category might be that the negative state enhances the subsequent auditory early processing regardless of sound frequency. Furthermore, the difference in pupillary responses between emotional conditions after the probe stimulus in the current task was smaller than that between deviant and standard tones. The effect size of the main effect of sound frequency condition was larger than that of the picture category condition, which supports this claim. This may be the reason that an interaction between presentation frequency and emotional conditions was not observed, as the differences between the standard and deviant response may have overshadowed the differences between emotional responses. Overall, our results suggest that the frequency of tone stimuli presentation is irrelevant for the pupillary response to tone in each emotional state.

In conclusion, we investigated the pupillary response to sound probe stimuli after emotional arousal. We found that the specific pupillary responses to tone stimuli were neutral in the early component, positive in the late component, and negative in the peak latency of the PDRs. In addition, the results of deconvolution, a high-resolution analysis of the pupillary responses, indicated that these results were not affected by the short inter-stimulus interval between the picture and sound presentation. Importantly, the frequency of sound presentation did not affect the pupillary response to tone stimuli for each emotional state. A limitation of this study is that the finding that the peak latency of the PDRs differs depending on the emotional valence is unprecedented and is only a speculation. Therefore, further studies in this regard are warranted. Another limitation is that the hit rates in the positive condition were lower than those in the other picture category conditions. The participants in this study were only Japanese. The bias in the evaluation is presumed to be due to the influence of cultural differences in the emotional arousal by IAPS pictures^31^. Taken together, our results support the idea that emotional stimuli can alter attention resource allocation, which is indexed by the level of activity of the locus coeruleus.

## Materials and methods

### Participants

Thirty-two students (23 males; mean age = 21.3 years; range = 20–24 years; SD = 1.14 years) participated in this experiment. All participants were students at the Toyohashi University of Technology. A power analysis using G*Power software (G*power 3.1)^32^ indicated that a sample size of 30 would achieve 80% power for a repeated measures design, given a medium effect size (*f* = 0.25) and α = 0.05. The power analysis focused on the interaction effect of picture category and tone type. Two participants were excluded due to equipment failure. All participants had a normal or corrected-to-normal vision and normal hearing based on self-reports. The experiment was performed in accordance with the Declaration of Helsinki, and all participants provided written informed consent before the experiment. The experimental procedure was approved by the Committee for Human Research at the Toyohashi University of Technology.

### Stimuli

The visual stimuli included 150 pictures (19° × 14.2°) selected from the International Affective Picture System (IAPS) database^11^, consisting of 40 neutral pictures (e.g., mushrooms, household objects, and neutral scenery) followed by the standard tone (mean valence/arousal = 4.997, 2.953), 40 negative pictures (e.g., snakes, guns, and human mutilation) followed by the standard tone (mean valence/arousal = 2.599, 6.052), 40 positive (e.g., babies, erotic couples, and delicious foods) pictures followed by the standard tone (mean valence/arousal = 7.402, 6.005), 10 neutral pictures followed by the oddball tone (mean valence/arousal = 5.043, 0.364), 10 negative pictures followed by the oddball tone (mean valence/arousal = 2.646, 6.117), and 10 positive pictures followed by the oddball tone (mean valence/arousal = 7.500, 5.979). The IAPS pictures presented in combination with the oddball tone differed from the pictures in the standard tone condition to avoid a decrease in response due to habituation to the emotional pictures. However, in each picture category condition, the valence and arousal values between the two groups (standard and oddball tone) were controlled to be equal. The IAPS normative ratings were used to select the emotional category of each picture. All pictures were converted to grayscale using MATLAB 2017b (The MathWorks, Natick, MA, USA), and the average brightness of all pictures (*Y* = 101.098) was unified using the SHINE Toolbox^33^. To reduce the pupillary light reflex, the brightness of the background (*Y* = 201.098) was higher than the average brightness of the pictures. The auditory stimulus was produced using the MakeBeep function in Psychtoolbox-3^34–36^. The sinusoidal tones consisted of a 1000 Hz low pitch tone and a 1500 Hz high pitch tone. The sampling rate of both tones was 22,050 Hz.

### Procedure

Stimulus presentation and timing were controlled using MATLAB 2014a (The MathWorks, Natick, MA, USA) presentation software (Psychtoolbox-3). The stimuli were displayed on an LCD monitor (ViewPixx/EEG, VPixx Technologies; screen resolution: 1920 × 1080 pixels, refresh rate: 120 Hz) and presented through headphones (SoundTrue around-ear headphones, BOSE) in a dark experimental room. The participant’s chin was fixed at a viewing distance of 600.0 mm from the monitor.

**Figure 5:**
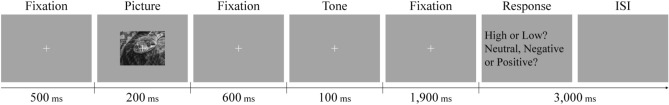
A diagram of the experimental procedure. In each trial, a fixation point was first presented for 500 ms. The International Affective Picture Set (IAPS) image was then presented for 200 ms. After the presentation of another fixation point for 600 ms, one of the two tones (standard or oddball) was presented for 100 ms. Each trial was separated by a response period and an inter-stimulus interval (ISI) of 3000 ms in total. Participants used the keypad to respond to the task in each block following the stimulus presentation.

Each participant performed 300 trials that were divided into 4 blocks of 75 trials to allow for short breaks. Figure 5 provides a visual representation of an experimental trial. Each trial presented stimuli in the following order: fixation point (500 ms), visual stimulus (200 ms), fixation point (600 ms), auditory stimulus (100 ms), fixation point (1900 ms), response period, and inter-stimulus interval (ISI) (3000 ms). The visual stimulus consisted of either a neutral (n = 100, 33.3%), negative (n = 100, 33.3%), or positive (n = 100, 33.3%) picture, presented randomly. The auditory stimulus was either an oddball (n = 60, 20%) or standard (n = 240, 80%) tone, presented randomly. For the odd-numbered participants, the low pitch tone was used as the standard tone, and the high pitch tone was used as the oddball. These assignments were reversed for the even-numbered participants so as to counterbalance any potential physiological response to the pitch of the tone itself. In addition, the oddball tones were set to never be presented continuously and to not be presented in the first and last trials of each session to further amplify the response to the oddball stimulus. At the end of each trial, the participants used the computer keyboard to respond, identifying the emotional categorization of the picture and pitch of the tone to ensure that they were paying attention to both stimuli. Possible response combinations consisted of a neutral picture followed by a low pitch tone (key response 2), neutral picture followed by a high pitch tone (key response 8), negative picture followed by a low pitch tone (key response 1), negative picture followed by a high pitch tone (key response 7), positive picture followed by a low pitch tone (key response 3), or a positive picture followed by a high pitch tone (key response 9). Participants were given a practice session and encouraged to practice until they memorized and were comfortable with the procedure, categorization task, and timing of the trial.

### Pupillometry

Pupil diameter and eye movement were recorded at all times during the task except for the response period and ISI. Pupil diameter and eye movement were monitored using an eye-tracking system (Eyelink 1000 Plus, SR Research) that consists of a video camera and an infrared light source pointed at the participant’s eyes and outputs the pupil size in arbitrary units. The pupil diameter was sampled at 500 Hz in the left eye only. At the beginning of each session, a nine-point eye-tracker calibration was performed. Participants were instructed to refrain from blinking as much as possible during the task.

### Data analysis

#### Behavioral data

The recorded pupillary and behavioral data were analyzed using MATLAB 2018a (The MathWorks, Natick, MA, USA). The behavioral data included the hit rates for the picture valence and the tone pitch. The hit rates for the picture valence were calculated by comparing the emotional valence categorized by the participant in the task with the emotional valence of the IAPS normative ratings (for example, whether the participant rated an IAPS image with a negative normative rating as a negative image). The hit rates for the tone pitch were computed by comparing the pitch categorized by the participant in the task with the correct pitch. The hit rates were computed for all the six response patterns separately for the picture (three patterns) and tone (two patterns) responses.

#### Pupillary data

The pupillary data during eye blinks were interpolated using cubic-spline interpolation^37^. Trials in which the pupils could not be detected during the beginning or end of the trial were excluded from the analysis. Next, subtractive baseline correction was applied for normalizing using the mean pupil size during the 200 ms before the auditory stimulus onset as a baseline^37^. Following the baseline correction, the pupillary data for each trial were smoothed using a moving-average filter with a 10 ms window and converted from the output of the arbitrary unit by the eye-tracking system to millimeters, based on a previous study^38^. Trials with additional artifacts, revealed by exceeding 0.011 mm/ms for the velocity of the pupil response, were excluded from the analysis. The average number of rejected trials was 39.83 ± 31.08 of 300 trials per participant. Finally, the grand-averaged pupil responses separated by picture category and tone type were computed. Tone type and picture category were set by the correct pitch and emotional valence based on the normative rating in IAPS. Previous studies have shown that the early and late components of the pupillary response to emotional pictures are different components that reflect the activity of the sympathetic and parasympathetic nervous systems, respectively^39^. Therefore, the early and late components of the PDR were measured as the average pupil diameter between 0–850 ms and 850–1700 ms, respectively, following the tone onset. Peak latency of pupillary response reflects many cognitive factors^40^, such as the frequency of stimulus presentation^41^. The PDR peak was defined as the largest pupil diameter observed within the 0–1700 ms following the tone onset, and PDR latency was defined as the duration between the tone onset and PDR peak.

In this study, the interval between picture and tone presentation was short (600 ms) and may have led to an overlap of the pupillary responses. Wierda et al. showed that it is possible to track attention and cognitive processes from slow pupillary responses (about 1 Hz) to high temporal resolution (about 10 Hz) by deconvolution of the pupillary response data^24^. The deconvolution approach assumes that each cognitive event is associated with an attentional pulse, which causes pupil dilation as a function of the intensity of the attentional pulse. Since pupillary responses are additive, we can predict the pupillary response pattern evoked by a task by convolving the attentional pulse with the pupillary response function, similar to the convolution process in functional MRI (fMRI) analysis. Therefore, we used this method to isolate responses to pictures and tones by calculating the average value of 200 deconvolution data. The interval between each pulse was 100 ms.

All statistical analyses were conducted using R software (version 3.5.3)^42^. Two-way ANOVAs were performed using picture category (neutral, negative, and positive) and tone type (standard and oddball) as within-subject factors. The level of statistical significance was set to p < 0.05 for all analyses. The pairwise comparisons for the main effects were corrected for multiple comparisons using the Modified Sequentially Rejective Bonferroni (MSRB) procedure^43^. Effect sizes (*partial η*^2^) were determined for the ANOVA effects. Greenhouse–Geisser corrections were performed when the results of Mendoza’s multisample sphericity test were significant. Statistical analyses for the pupillary index using deconvolution were performed for each time course with t-tests for standard vs. oddball tones and emotional (positive or negative) vs. neutral pictures for each pulse.

## Supplementary Information

Supplementary Information.

## Data Availability

The datasets generated and analyzed during the current study are available from the corresponding author on reasonable request.
