# Supplementary material for: Pupillary response reflects attentional modulation to sound after emotional arousal
Source: Sci Rep. 2021 Aug 26;11:17264. doi: 10.1038/s41598-021-96643-7 (PMC8390645; doi:10.1038/s41598-021-96643-7)
Supplement: Supplementary file 1 — Supplementary Information. [file 41598_2021_96643_MOESM1_ESM.docx]

Pupillary response reflects attentional modulation to sound after emotional arousal

Satoshi Nakakoga^1^*^a^*, Kengo Shimizu^1^*^b^*, Junya Muramatsu^2^, Takashi Kitagawa^3^, Shigeki Nakauchi^1^, Tetsuto Minami^1, 4*^

^1^ Department of Computer Science and Engineering, Toyohashi University of Technology, 1-1, Hibarigaoka Tempaku, Toyohashi, Aichi 441-8580, Japan

^2^ TOYOTA Central R&D Labs., Inc. System & Electronics Engineering Dept.II, 41-1, Yokomichi, Nagakute, Aichi 480-1192, Japan

^3^ TOYOTA MOTOR CORPORATION, R&D and Engineering Management Div., 1, Toyota-cho, Toyota, Aichi 471-8502, Japan

^4^ Electronics-Inspired Interdisciplinary Research Institute, Toyohashi University of Technology, 1-1, Hibarigaoka Tempaku, Toyohashi, Aichi 441-8580, Japan

*^a,b^* These authors contributed equally to this work.

*Corresponding author

E-mail: minami@tut.jp (TM)

We allow publishing the file provided in the supplementary material.

**Supporting information**

***・IAPS pictures***

The IAPS pictures (Lang et al., 2008) used in this study were: Neutral (Standard): 2190, 2191, 2200, 2214, 2215, 2396, 2749, 2840, 2850, 2880, 5130, 5500, 5531, 5534, 5740, 7000, 7002, 7004, 7006, 7010, 7020, 7034, 7038, 7080, 7090, 7096, 7100, 7150, 7161, 7175, 7185, 7207, 7211, 7217, 7224, 7233, 7493, 7595, 7710, 9070; Neutral (Oddball): 2102, 2221, 5471, 5532, 6150, 7009, 7140, 7283, 7950, 9210; Negative (Standard): 1019, 1090, 1201, 1220, 1525, 2120, 2683, 2691, 2717, 2981, 3100, 3120, 3130, 3150, 3170, 3180, 3230, 3250, 3500, 6020, 6210, 6242, 6313, 6410, 6540, 6821, 6838, 8231, 9040, 9140, 9250, 9253, 9410, 9421, 9429, 9433, 9495, 9594, 9902, 9921; Negative (Oddball): 1111, 1274, 2800, 3000, 3350, 5971, 6510, 8485, 9042, 9622; Positive (Standard): 1811, 2058, 2345, 4220, 4617, 4626, 4640, 4659, 4680, 4687, 5260, 5470, 5600, 5621, 5629, 5700, 5833, 5910, 7230, 7270, 7330, 8030, 8034, 8041, 8080, 8090, 8170, 8180, 8185, 8200, 8210, 8280, 8300, 8370, 8380, 8400, 8420, 8470, 8499, 8502; Positive (Oddball): 1710, 2208, 4290, 4660, 5460, 5480, 7400, 8190, 8501, 9156.
